# Comparative Effectiveness of Omeprazole and Lansoprazole in Gastroesophageal Reflux Disease (GERD): A Review Article

**DOI:** 10.31729/jnma.9021

**Published:** 2025-06-30

**Authors:** Rizky Gustinanda, Febby Zulkarnain, Merry Permatasari, Adonia Citra Farciana

**Affiliations:** 1 Faculty of Pharmacy, Ahmad Dahlan University, Campus III Soepomo, Warungboto, Umbulharjo, Yogyakarta, Postal Code 55164, Indonesia; 2 Faculty of Pharmacy, Sanata Dharma University, Campus III Paingan, Maguwoharjo, Depok, Sleman, Yogyakarta, Postal Code 55281, Indonesia.

**Keywords:** *drug effectiveness*, *gastroesophageal reflux disease*, *lansoprazole*, *omeprazole*

## Abstract

This review discusses the use of Omeprazole and Lansoprazole as therapy in patients with Gastroesophageal Reflux Disease (GERD). GERD is a digestive tract disorder caused by repeated reflux of stomach contents into the esophagus. GERD is often characterized by symptoms of a burning and burning chest (heartburn). The most widely used pharmacological therapy to treat GERD is Proton Pump Inhibitor (PPI) drugs, such as Omeprazole and Lansoprazole with a working mechanism that inhibits the proton pump that H+ ions exit from the gastric parietal cells. The method used in writing this article review is an electronic literature study by accessing national and international journal search sites. The results obtained are based on effectiveness, Omeprazole is more effective and faster in reducing and maintaining gastric acid pH. Based on the adverse effects of the drug, Omeprazole has a lower potential risk of causing diarrhea. Omeprazole has the most minimal cost analysis when viewed from the cost aspect and Omeprazole has a faster onset than Lansoprazole.

## INTRODUCTION

Gastroesophageal Reflux Disease (GERD) is a condition in which the contents and fluids in the stomach are constantly refluxed into the esophagus, triggering several indications of indigestion and complications in the stomach.^1^ Some clinical signs of GERD include burning in the chest (heartburn), stomach contents returning to the esophagus/ mouth (regurgitation), heartburn, nausea, sore throat when swallowing (odynophagia), nausea, difficulty swallowing (dysphagia), and difficulty sleeping at night.^2^ In general, GERD cases worldwide range from 15%-25% with Indonesia reaching 27.4%. GERD can be influenced by various risk factors such as smoking, obesity, alcohol consumption, stress, overeating, habits after eating immediately lying down, consuming food or drinks that are too acidic, and irregular diet.^3,4^

GERD that remains untreated with lifestyle improvements requires therapy with pharmacological agents.^5^ The types of drugs that can be used and given include antacids, histamine 2 receptor antagonists (H2RA), proton pump inhibitors (PPI), and sucralfate. Pharmacological therapy that is relied on in the management of GERD patients mostly uses PPI class drugs which are given based on empirical experience.^4^ Research shows that PPI therapy shows more powerful and superior results compared to H2RA therapy in relation to relieving symptoms of erosive esophagitis (EE) and non-erosive reflux disease (NERD).^6,7^ Lansoprazole and omeprazole drugs are the most widely used PPI class drugs. The method of action of this drug is by inhibiting the proton pump that H^+^ ions remove from gastric pariental cells, so that gastric acid secretion can be controlled again.^8,9^

Omeprazole is one of the earliest generation of PPI drugs that have become a mainstay in the treatment of acid reflux disorders. When compared to other class of agents such as H2RA, prostaglandin analogs, and anticholinergics, omeprazole is far superior in terms of safety, tolerance, and ability to suppress stomach acid.^10,11^ It inhibits the adenosine triphosphate H§/K§-ATPase pump of parietal cells, which is the final step of acid production. Omeprazole suppresses basal gastric acid secretion and stimulates meal-induced acid secretion. The inhibitory effect of omeprazole occurs rapidly within 1 hour of administration, with maximum effect occurring within 2 hours. The inhibitory effect lasts for about 72 hours after administration, followed by a return to baseline activity within 3-5 days. Omeprazole is extensively metabolised by the hepatic cytochrome P450 (CYP) enzyme system, primarily via the CYP2C19 and CYP3A4 isoenzymes. Omeprazole has a short half-life of 0.5-1 hour in healthy subjects and about 3 hours in patients with impaired liver function.^12^

Lansoprazole is a next-generation PPI class drug whose chemical makeup resembles that of omeprazole but contains additional fluoride on the pyridine ring. Lansoprazole is more lipophilic than omeprazole; consequently, it can penetrate cell membranes for the conversion of sulfonic acid and sulfonyl derivatives, thus creating a faster acid-inhibition effect. Furthermore, lansoprazole also does not cause reduced vitamin B_12_ intake in the body as reported in omeprazole users.^10,13^ Lansoprazole is the initial drug that is converted into the active form in the acid-secretory canaliculi of the parietal cells. As the inhibition is irreversible, acid secretion is suppressed for a period of 24 to 48 hours, until new proton- pump molecules have been synthesised and transported to the cell membrane.^14,15^

Omeprazole and lansoprazole drugs are similar both structurally and chemically, but there are relatively few comparisons of these drugs. The side effects of these two drugs also vary from patient to patient. There is no systematic review to compare the safety and effectiveness of omeprazole and lansoprazole. Therefore, the authors conducted this systematic review to identify study articles comparing the effectiveness and safety of omeprazole and lansoprazole in GERD.

## METHODS

The method used in writing this article review is to conduct an electronic literature search by accessing national and international journal search sites related to the keywords "Effectiveness of Lansoprazole and Omeprazole in GERD Patients". The inclusion criteria for the selection of journals used were journal publications that contained discussions of the keywords searched and published in the last 10 years (2014-2024). Of the 204 articles that appeared in accordance with the keywords, 6 journal articles were obtained on PubMed, NCBI (National Center for Biotechnology Information), and Google Scholar which were then used as literature in writing this review.

## RESULTS

Based on article searches that have been conducted, 6 out of 204 articles were obtained that can be used as reference literature for writing this article. Comparison of the use of Omeprazole and Lansoprazole drugs in patients with GERD can be seen in Table 1.

**Table 1:** Comparison of Omeprazole and Lansoprazole Drug Use in Patients with Gastritis

No.	Article Title	Research Methods	Research Results
**Effectiveness**			
1.	(16)	In this journal, the search was conducted systematically MEDLINE, EMBASE (from the beginning of the year to December 2019) and CENTRAL (January 2011-December 2019).	This article explains that omeprazole is faster to reduce the pH of gastric acid. In addition, omeprazole is better able to maintain the pH of acid gastritis (pH>4) and produces a longer-lasting effect than lansoprazole to control gastric acid.
2.	(17)	In this journal, the search was conducted based on literature in Medline (via PubMed) and Embase databases covering reports from 1985 to 2020.	The article explains that omeprazole should be taken before meals to maintain optimal gastric acidity (oral bioavailability = 30-40%). Whereas lansoprazole should not be given with meals, but rather just before meals rather than without meals to maintain optimal control of gastric acidity throughout the day (oral bioavailability = 80-85%).
3.	(18)	The research method uses a prospective observation research method.	The effectiveness of lansoprazole combined with antibiotics for the treatment of Hp positive gastric ulcer in adults is better and more effective than the use of omeprazole combined with antibiotics.
**Drug Side Effects**			
1.	(10)	The search was conducted through Pubmed, Pubmed Central, and Google Scholar databases by entering keywords such as Omeprazole, Lansoprazole, Clostridium difficile, Clostridium difficile infection. Journals were selected according to the predetermined inclusion criteria and exclusion criteria.	The use of high doses and for a long period of time in the drug Lansoprazole has a higher potential risk than the drug Omeprazole to increase Clostridium difficile infection. Lansoprazole increases the potential risk of Clostridium difficile infection by 83.8% while Omeprazole only has a potential risk of 55.8%.
**Cost**			
1.	(19)	This research uses a non- experimental method with a retrospective approach, then the data is processed using the Average Cost Effectiveness Ratio (ACER) method.	The cost of using PPI class drugs using Omeprazole is IDR 383,399.91 and Lansoprazole is IDR 667,321.019 so it can be concluded that Omeprazole has the least or minimal cost analysis.
**Onset**			
1.	(20)	The research design and procedure was a prospective study, with an open-label, two-way cross-over study.	Oral administration of omeprazole led to a rapid increase in intragastric pH, with the pH increasing significantly compared to lansoprazole at 3 hours after the start of pH measurement indicating that the onset of omeprazole drug effectiveness is faster even at the required once-daily use of the drug. Omeprazole administration showed a more favorable effect than lansoprazole administration on NAB (Nocturnal Gastric Acid Breakthrough). Omeprazole is more effective and faster to inhibit gastric acid secretion and can reduce NAB.

## DISCUSSION

GERD is a gastrointestinal disorder caused by the frequent backflow of stomach contents into the esophagus.^2^ This condition occurs due to the dysfunction of the lower esophageal sphincter (LES), which is the muscle ring at the bottom of the esophagus, between the esophagus and the stomach. The LES plays a crucial role in the digestive system. Its ability to open and close allows food to enter the stomach and prevents stomach contents from refluxing into the esophagus. GERD happens when the LES fails to function properly, either by not closing completely or opening at the wrong time, allowing stomach contents to flow back into the esophagus.^21^ The primary symptoms of GERD include a burning sensation in the chest (heartburn) and regurgitation. The most commonly prescribed medications for GERD are PPIs, a class of drugs that reduce stomach acid secretion. PPIs are among the most widely used treatments for GERD worldwide and are considered the most effective. Omeprazole and lansoprazole are the most commonly used drugs, having been available the longest. Despite their structural and chemical similarities, there have been relatively few direct comparisons of these medications.

Omeprazole and lansoprazole drugs are PPI and they work by reducing gastric acid secretion. According to Arezoomandi & Adibi (2019), it can be obtained that omeprazole reduces gastric acid secretion and heartburn symptoms significantly between 0-8 weeks during treatment with a p value <0.001.^22^ This is also supported by another study where it was reported that omeprazole showed a significant increase in pH compared to lansoprazole (p<0.05).^20^ In the results of literature study research from Javed et al. (2020) it was reported that omeprazole 40 mg provided a statistically significant increase in intragastric pH which was higher than lansoprazole 30 mg.^16^ The effectiveness of omeprazole is also supported from the study of Howden et al. (2009) who observed a long period of significantly higher gastric pH using omeprazole treatment compared to lansoprazole (p = 0.005). Although both drugs were well tolerated, the mean intragastric pH of omeprazole was higher compared to lansoprazole with a value of p = 0.003.^23^

Omeprazole is the most commonly used type of PPI, followed by Lansoprazole. Omeprazole and Lansoprazole in the last 20 years are in fact often used for therapeutic agents and given for a long period of time.^10^ This long-term and continuous use can reduce the production of the amount of acid in the stomach, as a result this can trigger a hypochloridia condition where the risk of being infected with enteropathogenic bacteria or parasites becomes higher, one of which is Clostridium sp.^24^ Clostridium sp. is a gram-positive bacterium that is shaped like an anaerobic rod and can produce exotoxins that are pathogenic. These bacteria usually act as saprophytes in the soil and digestive tract in humans. One of the pathogenic Clostridium sp. bacteria is Clostridium difficile. A person infected with this bacterium is believed to develop mild diarrhea with no limit to more serious problems.^25^

A study conducted by Imhann et al. (2016) proved that there was an increase in the number of patients infected with diarrhea due to Clostridium difficile in patients who used PPIs.^26^ This opinion is further strengthened by the results of research conducted by Bavishi & Dupont, (2011) which states that there is a relationship between the use of PPIs and an increase in enteric infections caused by Clostridium difficile.^27^ In addition, based on research conducted by Habibah et al. (2021), it was found that long-term use of Omeprazole on the increase in Clostridium difficile Infection (CDI) proved that Omeprazole has a relatively large potential (55.8%) in increasing C. difficile infection if used at high doses and for a long period of time. This is also reinforced by a case series study conducted by Nylund et al. (2014) which states that 30.08% of CDI cases were found due to long-term administration of omeprazole. Long-term use of omeprazole can affect gene expression in the gastrointestinal tract which can make the death of some secondary bacteria that have an important role in inhibiting the germination of sporative forms of C. difficile.^28^

This has been proven by a study conducted by Freedberg et al. (2015) on 12 volunteers who were given Omeprazole therapy for 8 weeks. This is different from the long-term use of Lansoprazole on the increase in CDI, which proves that Lansoprazole has a large potential effect (83.8%) in increasing C. difficile infection in the Gastrointestinal Track (GIT) system, if used over a long period of time.^29^ Research by Habibilah et al. (2021) proves that Omeprazole has a smaller effect than the drug Lansoprazole in increasing Clostridium difficile infection in causing diarrhea.^10^

According to research conducted by Funaki et al. (2013), the pharmacokinetic profile of PPI class drugs after postmeal administration may differ depending on the patient's condition. The inhibitory effect of PPIs on gastric acid secretion is known to correlate with the area under the blood concentration-time curve. Absorption of PPI drugs will affect the time of drug administration. A study on the determination of drug effects on intragastric pH through monitoring intragastric pH for 24 hours. The results showed that the percentage of intragastric pH holding time >4 for omeprazole drugs was 29.3 § 19.3-50.0% while for lansoprazole drugs was 27.8 § 13.0-42.3%. Intragastric pH >4 can be maintained significantly higher using omeprazole with a p value <0.05. Administration of omeprazole showed a more rapid increase in pH and could be seen at three hours after the start of pH measurements.^20^

Cost-effectiveness analysis (CEA) is a method applied to compare the relative costs and outcomes of two or more health interventions. In the AEB system, the achievement will be measured in non-monetary units (not in rupiah), such as the number of deaths prevented or a mmHg reduction in diastolic blood pressure.^30^ Based on research conducted by Pratiwi & Azzahra. (2022), it can be seen that the ACER value of omeprazole is smaller than lansoprazole. It is also seen from the number of successes and length of hospitalization that omeprazole provides higher effectiveness results compared to the effectiveness of lansoprazole.^19^ This effectiveness is inversely proportional to the length of hospitalization. This is also reinforced by research from Hutahaean et al. (2019) which shows that the ACER value of omeprazole is more cost effective than lansoprazole for gastritis therapy.^31^ Lansoprazole's ACER value is Rp 667,321,019 with an average length of treatment of 3.6 days. However, omeprazole only provides an ACER value of Rp 383,399.91 with an average length of treatment of 5.1 days.^19^

Although this study provides a comprehensive overview of the comparative effectiveness of Omeprazole and Lansoprazole in the treatment of GERD, there are some limitations that need to be noted. Firstly, this study was largely based on a literature review referring to secondary data, which means there may be variability in the methods and populations used in each of the included studies. This may affect the generalizability of the results, especially in different age groups, races, or specific medical conditions. Secondly, while there is data showing a comparison between the two drugs, there is no randomized controlled trial directly comparing the two with a more controlled research design. This reduces the ability to draw stronger conclusions regarding differences in the effectiveness and safety of the drugs in different conditions. In addition, this study did not fully explore the potential longterm side effects of using both drugs in a wider population, such as patients with comorbidities or elderly patients who may be more susceptible to side effects.

Suggestions for future research include conducting further studies with more robust experimental designs, such as randomized controlled trials (RCTs), which may provide stronger and more reliable evidence regarding the direct comparison between Omeprazole and Lansoprazole. Studies should also take into account individual patient factors, including age, gender, and other medical conditions, to evaluate how these variables affect the effectiveness and safety profile of the drugs. In addition, long-term studies looking at the side effects of using these two drugs, especially in relation to the risk of infection and disruption of the gut microbiota, are essential to provide a more comprehensive picture of their benefits and risks in GERD therapy.

## CONCLUSIONS

The results of this study show that Omeprazole is superior to Lansoprazole in several aspects related to the treatment of Gastroesophageal Reflux Disease (GERD). Omeprazole was shown to be faster in lowering and maintaining the pH of stomach acid, as well as having a faster onset of effect. In addition, the use of Omeprazole before meals is more effective in controlling stomach acid than Lansoprazole, which must be given just before meals. In terms of risk, high-dose and long-term use of Omeprazole has a lower potential risk of increased diarrhea-causing Clostridium difficile infection compared to Lansoprazole. In addition, Omeprazole is also more economical with lower cost than Lansoprazole. Overall, Omeprazole is considered more effective and safer for the management of GERD.
